# Three-dimensional reconstruction imaging by C-arm computed tomography accurately visualizes in-stent neointimal formation in patients with stent-assisted coil embolization

**DOI:** 10.3389/fneur.2023.1131061

**Published:** 2023-08-01

**Authors:** Masashi Kotsugi, Ichiro Nakagawa, Kengo Konishi, Haku Tanaka, Hiromitsu Sasaki, Takanori Furuta, Ai Okamoto, Kenta Nakase, Ryosuke Maeoka, Shohei Yokoyama, Shuichi Yamada, Hiroyuki Nakase

**Affiliations:** ^1^Departments of Neurosurgery, Nara Medical University, Nara, Japan; ^2^Division of Central Radiation, Nara Medical University, Nara, Japan

**Keywords:** 3D reconstruction imaging, neointimal formation, stent-assisted coil embolization, thromboembolic complication, cerebral aneurysm

## Abstract

**Background:**

Stent apposition to the vessel wall and in-stent neointimal formation after stent-assisted coil embolization for intracranial aneurysm are important factors associated with postoperative thromboembolic complications. No assessment methods have been established to depict 3-dimensional (3D) all-round in-stent neointimal formation.

**Objective:**

To demonstrate the superiority of Dyna-3D imaging assessment as a modality for all-round ISNF in comparison with conventional two-dimensional digital subtraction angiography (2D-DSA).

**Methods:**

Consecutive patients who underwent braided stent-assisted coil embolization for unruptured aneurysm between November 2016 and September 2021 were enrolled. Radiological assessments for stent apposition to the parent vessel after stent deployment and in-stent neointimal formation after 3 months were obtained. Dyna-3D was reconstructed by overlapping a plain image showing stent struts with a rotational DSA image showing the vessel lumen. Reconstructed Dyna-3D images can be rotated to any angle on the screen to evaluate to stent apposition around the vessel and in-stent neointimal formation in 3D, for comparison with 2D-DSA evaluations.

**Results:**

Among the 73 patients enrolled, 70 patients (96%) showed complete stent wall apposition on Dyna-3D. Higher intra-rater agreement was confirmed on assessment of in-stent neointimal formation with Dyna-3D (Cohen’s *κ* = 0.811) than with conventional 2D-DSA (Cohen’s *κ* = 0.517). in-stent neointimal formation could not be confirmed on conventional imaging in 9 cases (16%) and on Dyna-3D in 2 cases (3%). The number of in-stent neointimal formations rated as stent wire completely outside the endothelial line was significantly higher with Dyna-3D than with 2D-DSA (*p* = 0.0001).

**Conclusion:**

All-round 3D evaluation by Dyna-3D imaging appears useful for confirming in-stent neointimal formation after braided stent deployment in patients after stent-assisted coil embolization.

## Introduction

Stent-assisted coil embolization is a promising endovascular treatment for intracranial wide-necked aneurysm. However, stent deployment increases the risk of periprocedural thromboembolic complications and may result in serious ischemic neurological deficits compared with simple aneurysmal coil embolization (1, 2). Stent apposition to the parent vessel wall and in-stent neointimal formation after stent deployment have recently been recognized as crucial factors associated with periprocedural thromboembolic complications. Heller et al. reported that 36% of patients who had undergone stent-assisted coil embolization for cerebral aneurysms showed perioperative ischemic events due to incomplete stent apposition to the vessel wall (3, 4). Although accurate radiological assessment of stent apposition to the parent vessel and neointimal formation inside the stent is required to avoid periprocedural complications in the treatment of intracranial aneurysms, few reports have described in-stent neointimal formation in addition to stent apposition to the vessel wall using computed tomography (CT), magnetic resonance imaging (MRI), and digital subtraction angiography (DSA) (5, 6). Neointimal formation after stent-assisted coil embolization has recently been evaluated using optical coherence tomography (OCT) and optical frequency domain imaging (OFDI) (7, 8), but those intravascular imaging modalities are invasive when assessing intracranial stents.

Advances in imaging technology have enabled the acquisition of 3-dimensional (3D) volume-rendered images with C-arm systems at image qualities that allow visualization of stent apposition and creation of dual-volume 3D fusion images of 3D-DSA and high-resolution cone beam CT that allow detailed delineation of stents within the vasculature (6). In this study, we assessed neointimal formation in cases of braided stent-assisted coil embolization. This is because braided stents have four radiopaque tantalum markers on the proximal/distal ends with helical nickel-titanium (nitinol) wires within the body of the stent, facilitating visibility. The purpose of this study was to compare assessments of neointimal formation between 3D-Dyna and conventional 2D-DSA images and to confirm that 3D-Dyna images allow evaluation of “all-round” neointimal formation inside a braided stent.

## Methods

This retrospective observational study was based on the criteria of the STROBE (Strengthening the Reporting of Observational Studies in Epidemiology) statement. The institutional review board of our hospital approved this study (approval no. 2421).

### Inclusion criteria

**Figure 1:**
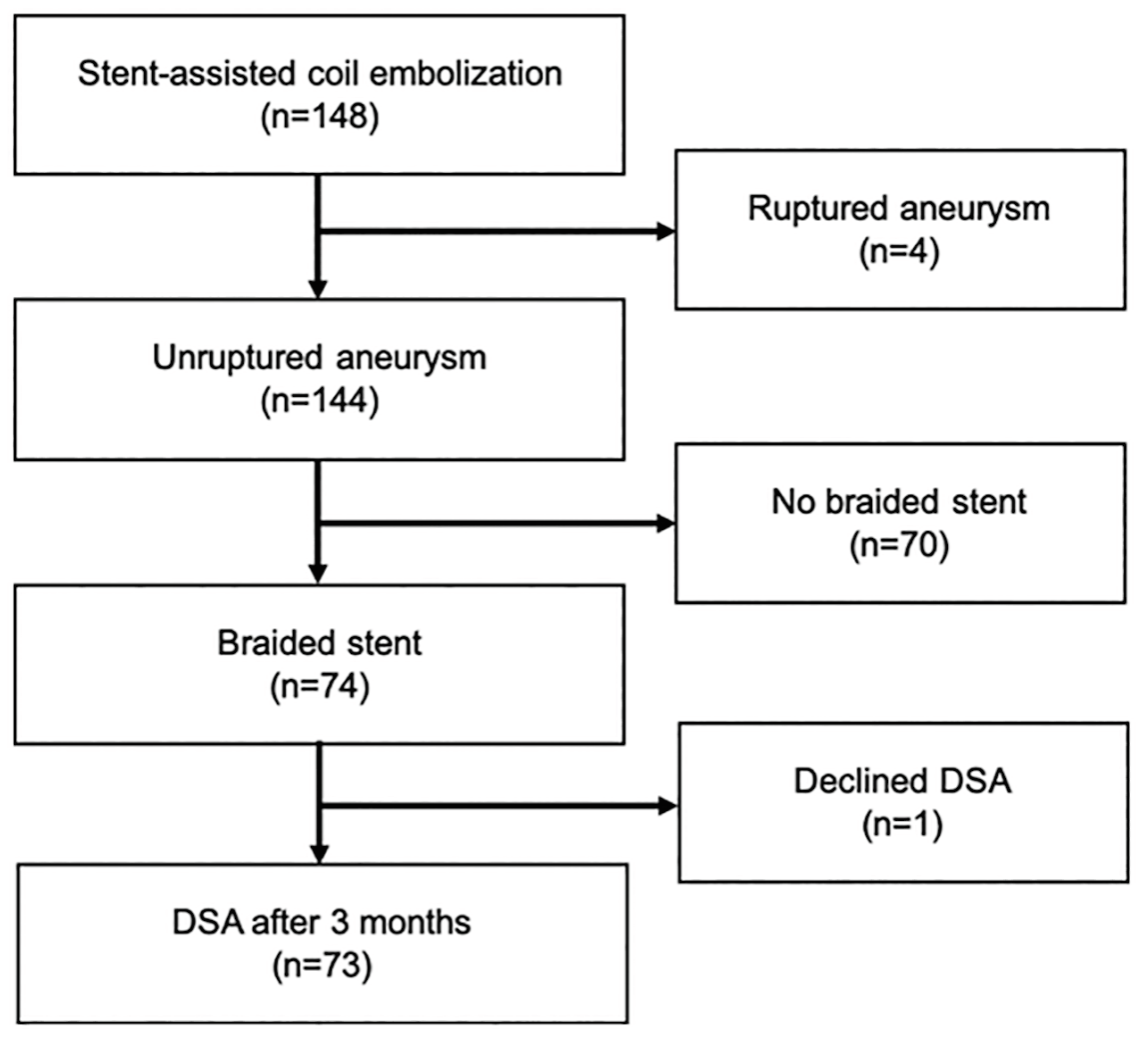
Flow diagram of the study.

Inclusion criteria comprised treatment of unruptured aneurysm between November 2016 and September 2021 by stent-assisted coil embolization using a braided stent, and performance of radiological assessments both just after coil embolization and 3 months after procedures. Patients with a history of aneurysm rupture, coil embolization with a non-braided stent, or unavailability of follow-up radiological assessments were excluded (Figure 1). A braided stent was applied for wide-necked aneurysm when the dome-to-neck ratio was <2.0 or neck length was >4 mm. Braided stents were used for internal carotid artery (ICA) and vertebral artery (VA) aneurysms for flow-diversion effects (9, 10).

### Interventional procedure in detail

At least 1 week before coil embolization, aspirin (100 mg/day) and clopidogrel (75 mg/day) were administered in all cases. Resistance testing for antiplatelet drugs was performed using VerifyNow (Accumetrics, San Diego, CA, United States). If the value of aspirin reaction units was <550 or the value of P2Y12 reaction units was <240, the aspirin dose was increased to 200 mg/day or cilostazol was added at 200 mg/day. All procedures were performed under general anesthesia with continuous monitoring of muscle motor evoked potentials. Unfractionated heparin was administered during the procedure to maintain an activated clotting time >250 s in all cases. A transfemoral approach was applied. A guiding catheter was advanced into the cervical portion of the ICA and a distal access catheter was advanced into the petrous portion of ICA for anterior circulation aneurysms. For VA aneurysms, a guiding catheter was advanced into the subclavian artery and a distal access catheter was advanced into the VA. A microcatheter for stent deployment (Headway 21 microcatheter; Terumo, Tokyo, Japan) was navigated to distal of the aneurysm and a microcatheter for coil embolization (Excelsior SL-10 microcatheter; Stryker Neurovascular, Freemont, CA, United States) was advanced into the aneurysmal dome. A braided stent (LVIS stent; Terumo, Tokyo, Japan) was deployed to cover the aneurysmal neck. To achieve sufficient expansion of the braided stent, stent crimping was optimized during stent deployment by controlling the movement of the microcatheter and the proximal pusher wire (9, 10). Coil embolization was performed via jailed microcatheter. Just after stent-assisted coil embolization, Dyna-3D angiography was performed and 3D reconstructed images were produced to evaluate stent wall apposition. One month after coil embolization, aspirin or clopidogrel were discontinued in cases with complete stent apposition to the vessel wall. Three months after treatment, 3D reconstructed images were again obtained to evaluate in-stent neointimal formation.

### Radiological imaging condition

**Figure 2:**
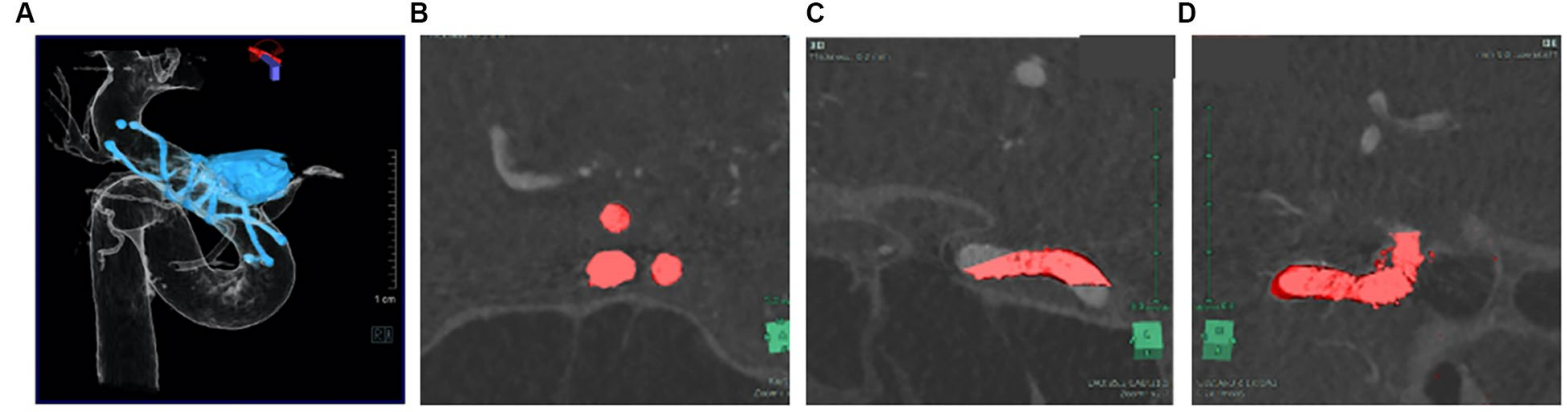
Plain image (stent, coil) and subtraction image (vessel) were overlapped VR images **(A)** that match the vascular cross section of MPR at all angles **(B-D)**.

MRI and time-of-flight magnetic resonance angiography (MRA) were performed the day after the procedure on 3 T scanners. A signal-hyperintense area on the ipsilateral side on diffusion-weighted imaging was defined as an ischemic lesion. Images were reconstructed and evaluated using 3D rotational angiography (3DRA). All cerebral angiography procedures were performed in an angiographic suite equipped with a flat-panel biplane system (Siemens Artis Q biplane; Siemens Healthcare, Forchheim, Germany). Angiography was performed via access using a 4-Fr transradial sheath. Undiluted contrast medium (300 mg/mL solution) was injected intra-arterially. Conventional DSA was followed by 3DRA with undiluted contrast (5 s acquisition, 2.5–3.5 mL/s contrast injection with delay time 1–1.5 s, Syngo Dyna-3D, Siemens Healthcare). Syngo Dyna-3D displays 3D projection images acquired with an angiography system. Plain imaging displays the coil mass and stent struts, while 3DRA displays the vessel lumen. Plain images (stent and coils) and subtraction images (vessels) were therefore overlapped (Figure 2A) and checked to ensure multi-planar reconstruction (MPR) images and volume rendering (VR) images were consistent between the SYNAPSE VINCENT medical imaging system (FUJIFILM Medical Solutions, Tokyo, Japan) and Syngo workspace imaging system (Siemens Healthcare). To avoid errors during 3D imaging reconstruction, the coordinates of MPR and VR images were adjusted in at least three directions (Figures 2B–D). Variability may be seen among creators in VR images. VR images that matched the vascular cross-section of MPR at all angles so that VR images matched the diameters of vessel lumens were used to resolve discrepancies among creators.

### Evaluation of angiography

**Figure 3:**
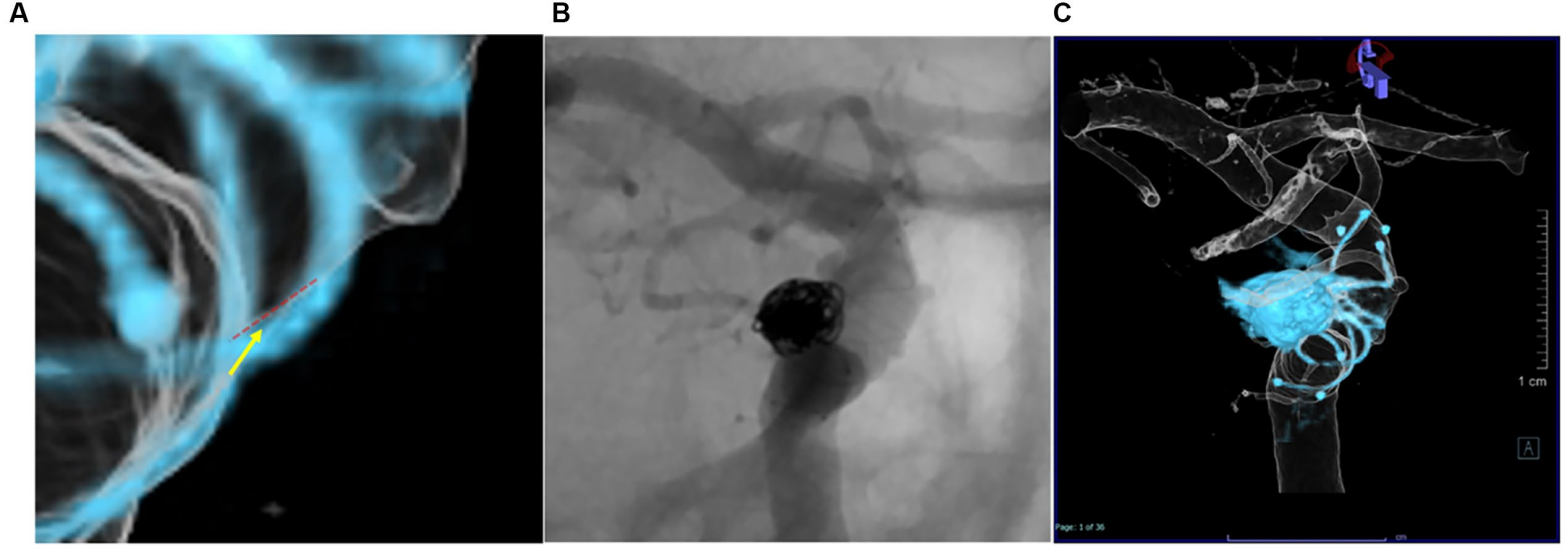
Intimal formation was defined as a Group A; The wire is completely outside the endothelial line, Group B: Wire overlaps the endothelial line, Group C: The endothelial line is outside the wire. The red dotted line is the endothelial line, and the yellow arrow is the wire line **(A)**. Differences in evaluation of neointimal formation between assessors using conventional 2D-DSA **(B)** and Dyna-3D **(C)**.

Stent apposition to the parent vessel was evaluated using 3D reconstructed images and Dyna-3DRA. All assessments were performed by two neurointerventionalists (MK, IN). Neointimal formation in the stent was visualized by 3D reconstructed images for the entire circumference. The implanted stent was divided into three areas (distal, middle and proximal) on 3D reconstructed images optimized with MPR images using a Syngo Workspace (Siemens Healthcare) workstation and on regular DSA images. Intimal formation was defined as stent wire completely outside the endothelial line (Figure 3A), and assessments of images in each area were performed by the same two neurointerventionalists (MK, IN). The degree of neointimal formation was classified into three groups: Group A: the wire is completely outside the endothelial line, Group B: wire overlaps the endothelial line, Group C: the endothelial line is outside the wire. If the wire partially overlaps the endothelial line as far as it can be confirmed, it is included in Group B in both 2D and 3D evaluations. The 2D images was magnified, and the longest possible stent axis was used to estimate the distance between the tantalum wire and the contrasted vascular lumen. The accuracy of visualization of neointimal formation was compared between 3D and conventional 2D images (Figures 3B,C). The relationships between evaluation of vessel wall apposition and neointima formation and postoperative thromboembolic complications were also examined.

### Statistical analysis

All statistical analyses were performed using IBM SPSS 26 (IBM Corp., Armonk, New York, United States). Neointimal formation was assessed and compared using the Wilcoxon test. An overall significance level of *p* < 0.05 was adopted. Concordance of the evaluation of neointimal formation between reviewers was confirmed using Cohen’s kappa statistic.

## Results

From November 2016 to September 2021, a total of 148 patients underwent stent-assisted coil embolization in our institute. Among these, 74 patients (12 men, 62 women; mean age, 59.6 years; range, 27–80 years) were treated using braided stents and were retrospectively reviewed. Postprocedural DSA was obtained after 3 months in 73 patients. The remaining patient declined to undergo postprocedural DSA (Figure 1). Table 1 shows a clinical summary of the patients. Of the 73 aneurysms, 47 (64%) were located in the ICA (C2–C3), 11 (15%) were in the ICA-posterior communicating artery, four (5%) were in the ICA below C4, nine (12%) were in the VA, and two (3%) were in the ACA. The most commonly used stent size was LVIS Blue (Terumo, Tokyo, Japan) 3.5 mm × 17 mm (51%). Dyna-3D shortly after the procedure confirmed complete stent wall apposition in 70 of the 73 patients (96%). In 2 of the 3 cases showing malapposition, the proximal edge of the stent exhibited incomplete apposition to the vessel wall. In the remaining case, stent apposition was unable to be evaluated because of a massive coil mass artifact. Postoperative ipsilateral DWI-bright lesions were observed in 35 cases (51%). No perioperative thromboembolic complications related to stenting were identified. However, one patient experienced partial visual disturbance due to occlusion of the central retinal artery after the procedure. No delayed ischemic or hemorrhagic complications were encountered during the observation period (range, 6–58 months).

**Table 1:** Summary of patients.

	Number (%)	Number (%)	
Age	59.6 (27–80)	LVIS Blue size	39 (51%)
Female	62 (84%)		
An. location		3.5 mm 22 mm	7 (9%)
ICA C2–C3	47 (64%)	4.0 mm 17 mm	11 (14%)
IC-Pcom	11 (15%)	4.0 mm 22 mm	1 (1%)
ICA below C4	4 (6%)	4.0 mm 28 mm	1 (1%)
VA	9 (12%)	4.5 mm 18 mm	9 (12%)
ACA	2 (3%)	4.5 mm 23 mm	1 (1%)
An. size		4.5 mm 32 mm	5 (7%)
<5 mm	4 (5%)	5.5 mm 33 mm	2 (3%)
≥5 mm, <12 mm	63 (86%)	Overlapping	
≥12 mm, <25 mm	6 (8%)	Single	70 (96%)
≥25 mm	0 (0%)	Double	2 (3%)
		Triple	1 (1%)
		DWI-positive	38 (52%)
		Ischemic complications	1 (1%)
		Delayed ischemic comp	0 (0%)

ICA, internal carotid artery; P-com, posterior communicating artery; An., aneurysm; LVIS, low-profile visualized intraluminal support device; DWI, diffusion-weighted imaging; comp., complication.

Table 2 shows differences in the visualization of neointimal formation between conventional 2D-DSA and Dyna-3D. Intra-rater agreement was higher with Dyna-3D (Cohen’s *κ* = 0.811) than with conventional 2D-DSA (Cohen’s *κ* = 0.517) (Figures 3B,C and Table 3). Neointimal formation could not be confirmed by conventional imaging in 17 cases (23%), or by Dyna-3D in 2 cases (3%). In the 2 cases for which neointimal formation could not be confirmed on Dyna-3D, confirmation was prevented by the coil mass. In these cases, one involved a fusiform aneurysm and the other involved recurrent giant aneurysm. The frequency of Group A findings was significantly higher with Dyna-3D imaging (*p* = 0.0001).

**Table 2:** Differences in visualization of neointimal formation between conventional 2D-DSA and Dyna-3D.

	Group A	Group B	Group C
Conventional-2D-DSA	26 (36%)	30 (41%)	17 (23%)
Dyna-3D	64 (88%)	7 (10%)	2 (3%)

Group A: excellent formation of neointima; Group B: good formation of neointima; Group C: poor formation of neointima. *p* = 0.0001.

**Table 3:** Assessment of the visualization of neointimal formation by each neurointerventionalist on Dyna-3D and conventional DSA.

		Dyna-3D (IN)			Total
		Group A	Group B	Group C	
Dyna-3D (MK)	Group A	63	1	0	64
	Group B	1	7	1	9
	Group C	0	0	0	0
Total		64	8	1	73

A, B intra-rater agreement is higher with Dyna-3D (Cohen’s *κ* = 0.811) than with conventional DSA (Cohen’s *κ* = 0.517).

## Representative case

**Figure 4:**
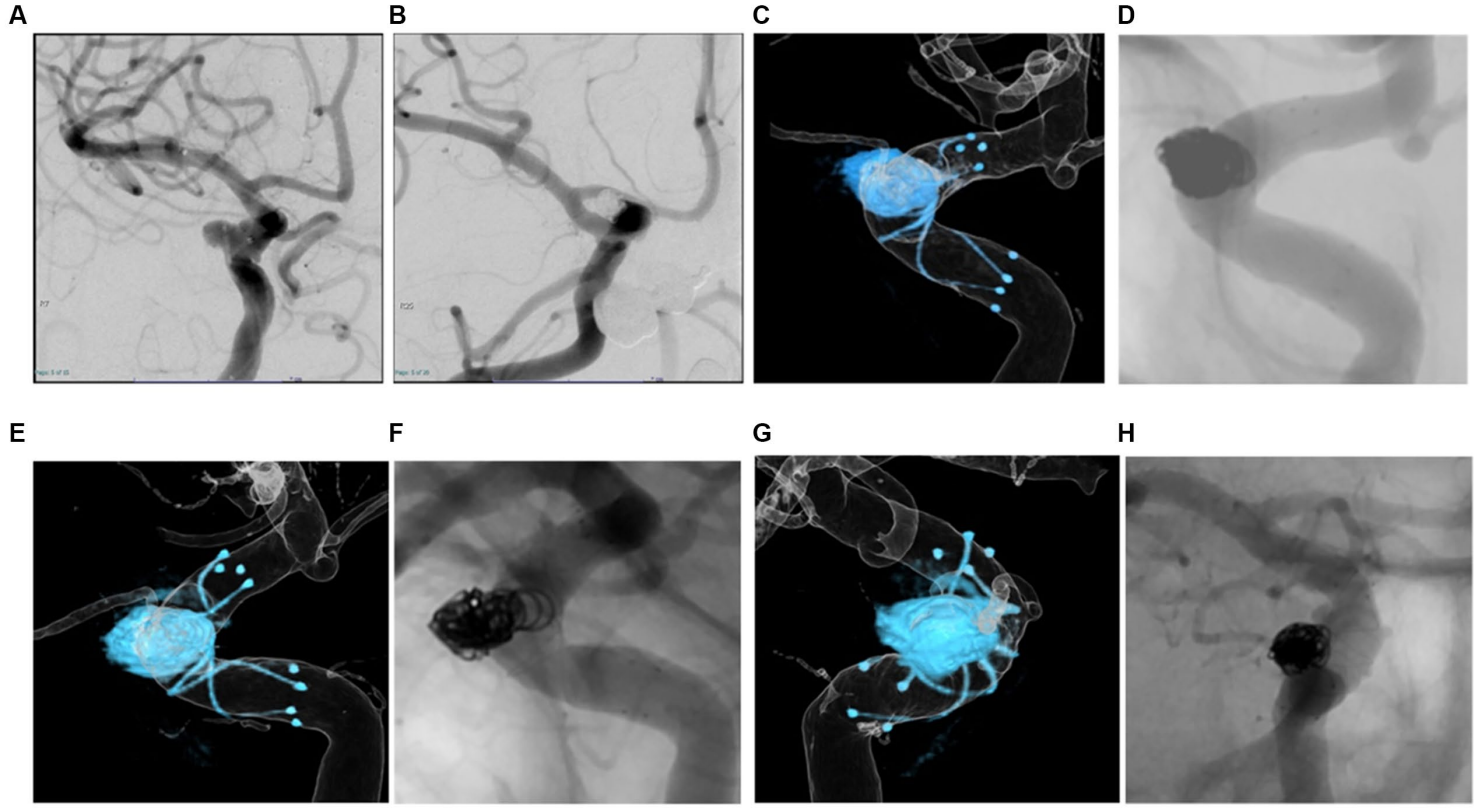
A patient in their 70 s. Braided stent-assisted coil embolization for internal carotid artery aneurysm was performed **(A,B)**. Dyna-3D image just after coil embolization shows tantalum wires of the stent inside the vessel lumen indicating appropriate stent apposition **(C)**. The conventional 2D imaging after just the procedure **(D)**. Three months after treatment, DSA shows tantalum wires of the stent outside the vessel lumen on conventional 2D imaging, but the image is unclear **(F,H)**. On Dyna-3D imaging, tantalum wires of the stent are clearly identified outside the vessel lumen over the entire circumference indicating excellent in-stent neointimal formation **(E,G)**.

A patient in their 70s underwent LVIS stent-assisted coil embolization of an unruptured aneurysm in the C2 portion of the right ICA (Figures 4A,B). Dyna-3D was performed just after coil embolization, showing the tantalum wires of the stent inside the vessel lumen (Figure 4C) and also conventional 2D imaging after just the procedure (Figure 4D). Three months later, DSA showed tantalum wires of the stent outside the vessel lumen on conventional 2D-DSA imaging, but the image was unclear and neointimal formation in the full circumference of the stent not be confirmed (Figures 4F,H). Dyna-3D imaging at the same time clearly showed the tantalum wires of the stent outside the vessel lumen (Figures 4E,G). This case was evaluated as Group B on conventional 2D-DSA and as Group A on Dyna-3D.

## Discussion

In this study, 3D evaluation using Dyna-3D after stent-assisted coil embolization was found to be highly useful for confirming in-stent neointimal formation and stent wall apposition over the entire circumference. Although stent apposition to the vessel wall has been assessed by CT, MRI, and DSA, the optimal method for precisely assessing stent apposition and in-stent neointimal formation of the vessel wall for the entire circumference has not been elucidated. In the evaluation of in-stent neointimal formation, we found that assessments using Dyna-3D were more consistent between assessors than those using conventional 2D-DSA.

The ischemic complication rate after coiling embolization with LVIS braided stents has been reported as 2.3%–6.5% (11–13). A braided stent has four radiopaque tantalum markers on the proximal and distal ends, with helical nitinol wires within the body. Therefore, unlike other neck-bridge stents, the position of the braided stent and the relationship to the inner wall of the parent vessel can be confirmed. On the other hand, a braided stent offers a high metal coverage ratio, requiring a skill to deploy with appropriate apposition to the vessel wall. Stent wall apposition just after deployment should therefore be checked, since poor neointimal formation due to incomplete stent apposition can delay in-stent thrombosis. A previous study analyzed stent wall apposition with post-treatment 3 T MRA and contrast-enhanced flat-panel CT (4). Postoperative MRI with gadolinium enhancement is another option to evaluate the stent structure and vessels surrounding the aneurysm (5). However, these modalities cannot be applied intraoperatively and provide only 2D slice images. In contrast, Dyna-3D can provide 3D images just after stent deployment and can be rotated freely to any angle to allow instant 3D evaluation of stent apposition around the vessel circumference. Furthermore, Dyna-3D can assess in-stent neointimal formation all around the parent vessel on 3 months follow-up DSA, providing additional information facilitating antiplatelet dose reduction and potentially preventing delayed thromboembolic complications. Kato et al. (6) reported dual-volume 3D fusion images obtained from 3D-DSA and a high-resolution cone beam CT allowed detailed delineation of stents within the vasculature. This modality holds promise as a tool for investigating stent apposition, but its applicability to evaluating neointimal formation inside the stent has not been previously examined. In 2 of 3 cases showing incomplete apposition, neointimal formation was unable to confirm on Dyna-3D. There are no studies for evaluating neointimal formation after placement of neck-bridging stent in the literature, however, it has been reported that incomplete stent apposition to the vessel wall occurs perioperative ischemic events since incomplete stent apposition may cause of poor neointimal formation (3, 4). Therefore, it would be clinically significant if neointima formation can be confirmed by radiological imaging as this finding gives reason to withdraw antiplatelet therapy as early as possible. The present study was able to confirm better imaging of the tantalum wires of the stent outside the vessel lumen on Dyna-3D compared with conventional 2D imaging. Further, Dyna-3D fusion imaging allowed more accurate visualization of circumferential neointimal formation, compared with conventional 2D imaging. Recent studies have reported evaluation of neointimal formation after stent-assisted coil embolization using OCT and OFDI (7, 8). However, such use of these modalities is invasive and requires insertion of a special catheter into the vessels. Dyna-3D does not need insertion of a special catheter into the stent, and so is less invasive than OCT. We therefore considered that Dyna-3D evaluation after stent-assisted coil embolization would be highly useful as a less-invasive modality for confirming neointimal formation within the entire circumference of the braided stent. Furthermore, future improvements in stent artifact suppression techniques could result in improvements in the quantitative, objective evaluation of neointima formation. If the metal artifact of the stent can be reduced, it may be useful not only for braded stents but also for the evaluation of the intimal formation of dense mesh stents.

The timing of evaluating neointimal formation is another critical issue. If neointimal formation is an indicator used to reduce the dose of antiplatelet drugs, checking for neointimal formation should be performed as early as possible. For intracranial stents, Lopes et al. (14) reported that the Neuroform stent (Boston Scientific Laboratories, Fremont, CA) showed complete intimal formation within the stent at 4 months after implantation. Previous studies have also demonstrated that re-endothelialization can take between 4 and 12 weeks for completion (15, 16). In the coronary arteries, neointimal formation within bare metal stents takes about 3 months (17). Based on such reports, we considered evaluation at 3 months after stent deployment as appropriate.

Perioperative administration of dual antiplatelet medications for stent-assisted coil embolization is the current standard in clinical practice, to reduce the risk of ischemic adverse events (18, 19). However, consensus remains lacking on the optimal duration of antiplatelet therapy after stent deployment. Discontinuation of antiplatelet therapy after Enterprise stent-assisted coil embolization is reportedly associated with ischemic complications, with a frequency of 3%–4.2% (20, 21). Incomplete stent apposition is known to result in poor neointimal formation and thus increases the risk of ischemic complications (3, 4). In a previous study, more than half of delayed thromboembolic events occurred within 1 month after switching from dual antiplatelet agents therapy (DAPT) to single antiplatelet agents therapy, regardless of the duration of DAPT (22). In addition to the ischemic complications associated with antiplatelet drug reduction or withdrawal, multiple antiplatelet therapy carries a risk of hemorrhagic complications (23, 24). The use of antiplatelet agents thus impacts the risk of hemorrhage. The hemorrhagic risk of stent-assisted coil embolization for unruptured intracranial aneurysm with DAPT is reportedly 2.2%–2.6% (25, 26), and delayed hemorrhagic complications may also occur (23, 24, 27). Discontinuation of antiplatelet medications as soon as feasible therefore appears prudent. On the other hand, to avoid delayed ischemic complications, antiplatelet drugs should be continued until neointimal formation within the stent is complete. With coronary stenting, current guidelines recommend continuing DAPT for at least 1 month after the implantation of bare metal stents. In the present study, cases with intimal formation evaluated as Group A or B on Dyna-3D showed no delayed ischemic complications, regardless of antiplatelet drug withdrawal at 3 months. These results suggest that Dyna-3D could represent an appropriate modality to identify neointimal formation in neck-bridge stents. As a result of this study, we could discontinue the use of antiplatelet medications as soon as possible to avoid delayed hemorrhagic complications and help mitigate short- and long-term sequelae. However, complete in-stent neointimal formation has not been confirmed to significantly suppress the incidence of delayed complications, and further studies are required.

### Limitations

Several limitations concerning evaluation using Dyna-3D need to be kept in mind. First, coil mass artifacts make the evaluation of large or fusiform aneurysms difficult. Metal artifacts cannot be completely removed and this remains a difficult issue for the future. Second, certain discrepancies may be encountered during 3D fusion image reconstruction between radiographers. At the time of VR imaging, the volume for VR should be matched on MPR to prevent overestimation for lumen of vessel. Third, some difficulties remain with the quantification of intimal formation on each modality. We instead defined intimal formation as the wire is completely outside the endothelial line. Cloud map was not used for comparison of intima formation in this study. This would have made the assessment more accurate. Fourth, this study only reported on a small number of cases from a single institution, with a short duration of follow-up. Finally, this report was retrospective in design.

## Conclusion

Three-dimensional evaluation using Dyna-3D after coil embolization with a braided stent appears highly useful for confirming neointimal formation over the entire circumference of the stent.

## Data availability statement

The raw data supporting the conclusions of this article will be made available by the authors, without undue reservation.

## Ethics statement

The studies involving human participants were reviewed and approved by Nara Medical University Hospital. The patients/participants provided their written informed consent to participate in this study.

## Author contributions

MK and IN conceived of the study, analyzed and interpreted the data, drafted the manuscript, and edited the manuscript. All authors contributed to the article and approved the submitted version.

## Conflict of interest

The authors declare that the research was conducted in the absence of any commercial or financial relationships that could be construed as a potential conflict of interest.

## Publisher’s note

All claims expressed in this article are solely those of the authors and do not necessarily represent those of their affiliated organizations, or those of the publisher, the editors and the reviewers. Any product that may be evaluated in this article, or claim that may be made by its manufacturer, is not guaranteed or endorsed by the publisher.

## Abbreviations

ISNF: in-stent neointimal formation
SACE: stent-assisted coil embolization
3D: 3-dimensional
ICA: internal carotid artery
MPR: multi-planar reconstruction
VR: volume rendering
